# Virtual controls as an alternative to randomized controlled trials for assessing efficacy of interventions

**DOI:** 10.1186/s12874-020-01191-9

**Published:** 2021-01-05

**Authors:** Joseph M. Strayhorn

**Affiliations:** Organization for Psychoeducational Tutoring, 205 Willard Way, Ithaca, NY 14850 USA

**Keywords:** Virtual controls, Nonrandomized, Observational, Randomization, Intervention research, Research design, Statistically generated controls

## Abstract

Randomized controlled trials are ubiquitously spoken of as the “gold standard” for testing interventions and establishing causal relations. This article presents evidence for two premises. First: there are often major problems with randomized designs; it is by no means true that the only good design is a randomized design. Second: the method of virtual controls in some circumstances can and should replace randomized designs.

Randomized trials can present problems with external validity or generalizability; they can be unethical; they typically involve much time, effort, and expense; their assignments to treatment conditions often can be maintained only for limited time periods; examination of their track record reveals problems with reproducibility on the one hand, and lack of overwhelming superiority to observational methods on the other hand.

The method of virtual controls involves ongoing efforts to refine statistical models for prediction of outcomes from measurable variables, under conditions of no treatment or current standard of care. Research participants then join a single-arm study of a new intervention. Each participant’s data, together with the formulas previously generated, predict that participant’s outcome without the new intervention. These outcomes are the “virtual controls.” The actual outcomes with intervention are compared with the virtual control outcomes to estimate effect sizes. Part of the research product is the prediction equations themselves, so that in clinical practice, individual treatment decisions may be aided by quantitative answers to the questions, “What is estimated to happen to this particular patient with and without this treatment?”

The method of virtual controls is especially indicated when rapid results are of high priority, when withholding intervention is likely harmful, when adequate data exist for prediction of untreated or standard of care outcomes, when we want to let people choose the treatment they prefer, when tailoring treatment decisions to individuals is desirable, and when real-world clinical information can be harnessed for analysis.

## Background

Testing the efficacy and safety of interventions in medicine, psychology, education, and other human services is of paramount importance. This article makes two interlocked claims about the methods for such tests. First: although randomized designs for testing medical, psychological, and educational interventions are ubiquitously referred to as the “gold standard,” there are often major problems with these designs; nonrandomized designs are often preferable or indeed the only ones possible. Second: a nonrandomized method called “virtual controls” can often provide a good alternative, especially in the presence of cooperative and cumulative research efforts to support it.

Much has been written on the pros and cons of randomization; this article will not attempt a systematic review. Rather, Part A of this article will briefly summarize the advantages of randomization, and then present evidence and reasoning about the major problems with randomized designs. Since randomized designs are so widely viewed as the gold standard for science, and since their advantages are standard subject matter for textbooks of statistics and research design, I will not give equal time, but explore thoroughly the disadvantages of random assignment.

Part B will rely upon logic and reasoning, as well as the presentation of empirical findings, to explicate the rationale behind the method of virtual controls. It will then provide some examples of the use of this method in research so far.

## Main text

### Part A: Problems of randomized designs

#### Internal and external validity

In intervention research, “internal validity” refers to the ability of a research design to establish that the intervention, and not some other lurking variable, caused the observed effect on the outcome variable. Persuasive logic supports the internal validity of randomized designs. The case for randomization in scientific inference was forcefully made by the statistical pioneer, R.A. Fisher, in 1925 [1]. A standard first year statistics text [2] told students: “How can a direct causal link between x and y be established? The best method – indeed, the only fully compelling method – of establishing causation is to conduct a carefully designed experiment…. Randomization … is an essential ingredient for a good experimental design.” (p. 235, p. 295) The Cochrane Group’s “Study Quality Guide” [3] defined a hierarchy of study designs, with randomized trials at the top and case series at the bottom. A director of the U.S. government’s Institute of Education Sciences stated, “Randomized trials are the only sure method for determining the effectiveness of education programs and practices.” [4] (p.6) From a review of pros and cons of randomized designs [5]: “According to the ‘Classical EBM [evidence based medicine] ideology’ the role of RCTs [randomized controlled trials] is beyond any question, which is why Sackett et al.… [6]. recommended: ‘if you find a study was not randomized, we’d suggest that you stop reading it and go on to the next article.’ “(p. 108).

The chief advantage of randomized designs is the quality of the “counterfactual” that they provide. The ideal strategy of causal inference would be to compare what happens when a causal agent exerts its effects (the factual) with what would have happened in identical circumstances, to identical people, at the identical time, if the causal agent had not been present (the counterfactual). Given that it is not possible to rewind time, we can never find out what would have happened with that imaginary ideal counterfactual. When people are randomly assigned to treatment versus control, the logic is that in the absence of a treatment effect, the control group, although not identical to the treatment group, would differ only to degrees predictable by the probability models of random assortment. That is: it is always possible that observed difference between outcomes with different treatment conditions are not caused by the treatment, but by selection factors. For example, one group happened to be healthier, or to have some preexisting characteristic that destined them to be healthier. In other words, bias in random selection into the groups is possibly responsible for the differences. But for any given observed difference, the probability of obtaining such a difference (or a greater one) from selection bias in the randomization goes down as sample size goes up, in ways that can be calculated. The beauty of randomized designs is that such logic applies to all ways in which the groups can be different: to those variables we know and can measure, and to those we have not thought of or can’t measure.

It’s worth noting that our inferences about the relative strengths of experimental designs spring from thought and logic, not from accumulation of data points. In 1963 Campbell and Stanley [7] enumerated possible threats to valid inferences, and explicated how different experimental and quasi-experimental designs take care of those threats; such an analysis is still widely accepted (e.g. Yu [8]) -- and rightly so. Thought experiments are very valuable in the study or research design.

As Campbell and Stanley noted, internal validity does not accomplish the whole job of research design. Showing that an intervention had an effect in the research sample does little good if the results cannot be generalized to people outside the study. For ideal generalizability, also known as external validity, statistical textbooks tend to specify as conditions for inference that the sample was randomly selected from a population to which one wishes to generalize. Random selection of the entire sample is, of course, distinct from random assignment to treatment group. How often are medical, psychological, or educational studies conducted by randomly selecting people from the population and successfully conscripting all those randomly selected, before then randomizing them to treatment groups? This happens approximately never. Because participants need to consent, and because researchers lack totalitarian power, intervention studies are almost always convenience samples.

Suppose a randomized trial of an intervention is carried out with people of average age 45, body mass index mostly in healthy range, average income upper middle class, mostly males, without asthma. A certain effect size is found. A clinician is treating a female, age 65, very low income, moderately obese, with moderate asthma. If we assume that the variables I mentioned interact with the intervention effect, we are hard put to predict an effect size for this individual. Is the expected effect even positive?

Adding to the generalization problem of randomized trials is that the people willing to submit to randomization are by definition a select group. The search term “volunteer bias” locates studies on this effect, for example that of Jordan et al. [9] who note that “non-targeted recruitment in all research designs favours healthier, wealthier, better educated, non-smokers, risking volunteer bias.”(p.2).

The sorts of people who are available for randomized trials can change over time. For example, researchers hypothesize that people with uncomplicated bipolar disorder signed up for trials of lithium, shortly after lithium became available. But as time went by, the bipolar patients who were quick and robust responders to lithium or other medications were successfully treated by clinicians, and those who sought research interventions began to have more complicated and treatment-refractory conditions [10]. Thus the effect sizes derived from randomized trials may change over time.

At least partly because of these external validity problems, the results of a substantial fraction of randomized trials have contradicted previous trials using the same methods, leading to questioning of the “reproducibility” of results. Niven and colleagues [11] studied 66 randomized trials in critical care medicine where there were reproduction attempts. “More than half of clinical practices with a reproduction attempt demonstrated effects that were inconsistent with the original study (56%, 95% CI 42–68%), among which a large number were reported to be efficacious in the original study and to lack efficacy in the reproduction attempt (34%, 95% CI 19–52%). Two practices reported to be efficacious in the original study were found to be harmful in the reproduction attempt.” (p.1) These authors cite five other studies suggesting “that less than half of reproduction attempts report results that are consistent with the original study.”(p.2) Ioannidis, [12] who examined highly cited medical articles, found that 9 of 39 replications of randomized trials found contradictory results or weaker effect sizes than initially reported.

We thus must admit that our “gold standard” research design, while being the best possible at demonstrating causality with the group of research participants we have assembled, is much less dependable at providing inferences extending to people outside the study. And making inferences outside the research sample is, of course, the purpose of research.

#### Ethics and equipoise

Suppose there is a life threatening illness, for which a new treatment or preventive has been devised. Enough evidence accumulates to give a team of investigators a high subjective probability that the intervention is quite effective. Then large numbers of people are randomized to active versus placebo intervention. The new intervention works, and there are far fewer deaths and far less morbidity in the treated group. The experiment is a great success. Everyone is happy -- everyone, that is, except the people randomized to placebo, who died or suffered permanent morbidity, and anyone who cares about them. They have sacrificed so that science could establish the usefulness of the treatment. We must ask: were their deaths really necessary? What if a good enough counterfactual could have been obtained from the detailed histories of those people who did not receive the treatment because it was not available yet? If “good enough” proof can be obtained without using placebo, lives may literally be saved. Research design takes on life and death importance.

It is a matter of debate as to when active controls, for example “standard of care,” must be used instead of placebo, and revisions of the Declaration of Helsinki have sought to update current ethical thinking [13]. But whether the comparison group is placebo or the best active treatment available to date, the hopes and expectations of the investigators usually are that the comparison subjects will have worse outcomes than the group receiving the innovative treatment.

Randomized trials have been said to pose no ethical dilemma when there is “equipoise,” i.e. when the subjective probabilities of benefit from treatment and control interventions are the same. But it usually impossible to convince funding sources to invest in very expensive clinical trials when there is literally no reason to expect that the treatment will be better than the comparison. Put another way, equipoise is usually not a good selling point in a grant proposal. It has been argued that researchers can ethically withhold active treatment from a randomly selected group when they believe the treatment is efficacious but the larger clinical community is in equipoise and needs to be convinced [14, 15]. But the investigators are often much more highly informed about the intervention than the community that needs convincing; the relative ignorance of the community, arguably, does not absolve investigators from responsibility for withholding treatments that they themselves strongly believe to be life-saving or permanently life-altering for the better.

The least ethically questionable practice exists when investigators, as clinicians, can recommend to every research participant the treatment the investigator believes to be best for that person. I will later argue that the method of virtual controls is compatible with this practice.

##### Demoralization as a threat to both internal validity and ethics

It is often difficult or impossible for research participants to remain blind to their random assignment, as for example in psychotherapy research, intubation, major surgery, or most educational interventions. Even in studies where blind conditions are attempted and maintenance of the blind is measured, research subjects have often been shown to guess correctly their assignment [16].

When participants are not blind to their treatment condition, randomization does not necessarily equalize a very important confounder: the degree of participant satisfaction with the outcome of randomized assignment. Dissatisfaction with one’s random assignment has been indexed by the term “resentful demoralization” (e.g. [17]). Suppose that 100 people sign up for a drug-versus-psychotherapy study, and 80 of them prefer the drug and 20 prefer therapy. If they’re divided into equal groups, we’d expect about 40 of the those who prefer drug and 10 who prefer therapy to be assigned to each group. Thus the drug group would have 80% of participants pleased with their assignment, whereas the therapy group would have 80% displeased with their assignments. This would be a very important source of bias. And even if one should recruit from the outset equal numbers of people preferring each treatment, dissatisfaction with assignment may affect the two interventions differently. Recruiting a sample with equal numbers preferring each treatment might be very difficult: if a treatment is promising, for example, it would probably be difficult to recruit participants preferring placebo.

On the other hand, if participants are allowed to select their own form of treatment, their degree of enthusiasm and optimism about treatment is more likely to be similar among groups. Thus, contrary to prevalent thinking, a non-random design allowing patients to follow their preference should equalize expectancy effects more effectively than randomly assigning them. In other words, the superiority of randomized designs for internal validity may disappear for interventions that cannot be delivered in blind fashion.

Allowing participants, in partnership with their clinicians or very informative investigators, to select their own treatment is relevant to ethics as well as to internal validity. If the fraction of participants receiving each intervention is held constant, and if expectancy effects do influence outcome, the outcomes for research participants as a group should be more favorable if participants can choose their interventions.

Regarding this point, I must refer to my personal experience with the real people who take part in research studies. On several occasions I have told a person about a study of a promising intervention, and have witnessed, along with the person’s enthusiastic signing of consent forms, the arousal of hope. I have then been present at the moment when the person was informed that they (or their child) had been randomized to the control condition. The memory of these moments strongly motivates the study of alternatives to randomization.

#### Time, labor, and expense of randomized trials

Manipulating treatments in randomized studies requires several time consuming steps. The ethics of withholding treatment from control participants needs to be carefully reviewed by an Institutional Review Board. Recruiting patients who are willing to be randomized takes time. The recruitment of 140 patients for a randomized study on depression in diabetic people [18] required contacting 18,925 people; that’s about 135 contacts per enrollee. Recruitment cost an average of $1358 per enrolled patient. Researchers in randomized trials must carefully explain the study to each candidate -- the concept of randomization is sometimes not easy for participants to grasp. The available person-power to do assessments and provide treatment is limited to research staff and collaborators. A 2008 examination of 28 randomized trials reported an average cost of 12 million US 2004 dollars per trial [19].

Meanwhile, every day, in clinical and educational settings, people are receiving interventions and data are being entered into records. A minute fraction of these data contribute to our accumulated scientific knowledge. Routine medical records may not contain predictor and outcome variables measured accurately enough to use nonrandomized designs, without the infusion of additional person-power to supplement the work of clincians. But the economics of supplementing and cleaning up clinical data through additional quality control labor and personnel still may be quite advantageous relative to randomized trials.

#### Time limits for random assignment

Randomized studies typically last a few weeks to a few months. Examples are found in US National Institute of Health multisite randomized intervention studies for child mental health. The CAMS (Child/Adolescent Anxiety Multimodal Study), [20] for example, randomized participants to groups receiving sertraline, cognitive behavior therapy, the combination, and placebo. The separation into the these groups lasted for 12 weeks; after that, placebo nonresponders were offered a choice of active treatments, and nonresponders to active treatments were referred to community providers – thus the adherence to randomly assigned conditions was ended. Why not maintain the separate groups for years of follow-up? One reason is ethics: 12 weeks is less troubling than several years of withholding alternative treatment from nonresponders. The second reason is practicality: even if one wanted to keep nonresponders from pursuing other treatments, in a free society they would still have the right to do so.

Other studies had similar time limits for sticking to randomized groups. For the TADS (Treatment for Adolescents with Depression Study) [21] separation was ended for one group after 12 weeks and for all groups after 36 weeks. The POTS (Pediatric OCD Treatment Study) [22] also maintained the separation of groups for 12 weeks.

But, to continue to refer to these examples, anxiety, depression, and obsessive-compulsive disorder are notoriously chronic conditions. For many people with these conditions, 12 weeks is only the beginning of years of treatment. The randomized trials studied the effects of putting the participants on serotonin reuptake inhibitors but did not study the effects of eventually taking them off these medications. Significant withdrawal effects of these medications have been reported [23]. What sort of intervention makes people best off after 5 or 10 years, or longer? If we are to “stop reading” studies that are not randomized, it is likely that we can never read the answer to such long-term questions.

On the other hand, if our research design lets people assort themselves into treatment groups according to their own preference, we eliminate the time pressure to end the randomized design. We have to deal with vicissitudes and vagaries of their choices, but at least we can follow them indefinitely without abandoning the original design.

#### Track record for randomized versus nonrandomized research

Teachers of research methodology (including myself) are emphatic that “correlation does not imply causation” (which is why, for example, even if basketball ability is significantly correlated with shoe size, coaches of sixth grade basketball teams should not outfit all their players with size 16 shoes). But when correlation is found and evidence renders rival causal hypotheses implausible, important and useful causal inferences have been made without randomization.

Several studies have attempted to use empirical methods to to compare the results of randomized and nonrandomized designs. A 1982 study by Sacks [24] concluded that “historical controls” seemed to have worse outcomes in the control group than in randomized trials, and that the bias this introduced appeared not to be correctible statistically. Subsequent studies have differed. Notably, Concato et al. [25] reviewed 99 studies on 5 clinical topics, comparing the estimated effect sizes of randomized versus observational (case-control and cohort) studies. The conclusion was that “the average results of the observational studies were remarkably similar to those of the randomized, controlled trials.”(p. 1887) The authors concluded that “The popular belief that only randomized, controlled trials produce trustworthy results and that all observational studies are misleading does a disservice to patient care, clinical investigation, and the education of health care professionals.”(p. 1892).

A famous example buttressing the necessity for randomization is the research done on estrogen supplementation for postmenopausal women, in which a randomized trial, the Women’s Health Initiative, [26] came to a different conclusion than a prior observational study, the Nurses’ Health Study [27]. However, Hernan et al. [28] reanalyzed the results of the observational study, incorporating analytic methods used in the randomized study, and stratifying on the time since menopause at which estrogen therapy was begun. In the reanalysis, “much of the apparent WHI-NHS difference disappeared….” (p.8).

Nonrandomized research designs are widely accepted in the study of harmful agents. An example of overzealous rejection of nonrandomized research came from the man who is probably the most influential statistician of the twentieth century, Ronald A. Fisher. After several landmark case-control studies of cigarette smoking and lung cancer had been published, e.g. by Doll and Hill [29], and the dangers of smoking were being publicized, in 1958 Fisher [30] wrote, “What is not so much the work of a good citizen is to plant fear in the minds of perhaps a hundred million smokers throughout the world … without knowing for certain that they have anything to be afraid of in the particular habit against which the propaganda is to be directed.” (p. 152) Fisher justified this contention on the grounds that “Replication…is not sufficient without the added precaution of randomization.”(p 153).

To this day, there has never been a study randomly assigning human beings to long-term use of cigarettes. But the idea that smoking causes lung cancer (and numerous other maladies) is not open to doubt.

Rare but serious side effects usually require nonrandomized research. Typically randomized trials bring drugs to market, but when the drug needs to be withdrawn from the market, postmarketing nonrandomized research is to thank. For example, a review of nine randomized trials of valdecoxib came to conclusions favorable to the drug [31]. Observational research and calculations of its risk of Stevens-Johnson syndrome and toxic epidermal necrolysis, among others, led to its withdrawal from the market [32]. For another example, a vaccine against swine flu was widely tried in the 1970’s; its use was criticized as a “fiasco” [33] and an example of rushing a vaccine to market before sufficient research was done, because it appeared to cause Guillaine-Barre syndrome. But the best estimates for the rate of increase in Guillaine-Barre cases were around 1 in 100,000 vaccinations [34] -- a rate that randomized trials with even 15,000 per group are not powered to detect. For rare but serious side effects of interventions, we tend to rely on nonrandomized observational research, because the sample sizes required can be larger than those feasible for randomized trials.

There is little doubt about the harmful effects of lead ingestion, multiple blows to the head, repeated sunburns, obesity, physical and sexual abuse of children, the teratogen thalidomide, glue-sniffing, and countless other noxious agents. No Institutional Review Board would accept a proposal to verify these propositions by randomly assigning the harmful factor. Nonetheless, science works around this constraint to produce accepted causal inferences. There is no logical reason why non-randomized research cannot do the same with therapeutic or preventive agents.

#### Summary of reasons not to sanctify randomization

The idea that the only good study is a randomized study is rejected for several sets of reasons: 1. The problem of generalizability, and associated problems with reproducibility of results. 2. Ethical questions and demoralization-related confounds resulting from purposely withholding treatment that is hoped to be very useful. 3. The time, effort, and expense of randomized trials, juxtaposed with clinical data that should not be wasted. 4. Time limits on how long random assignments can be maintained, juxtaposed with the chronicity of conditions whose treatments are studied. 5. A positive track record for nonrandomized research, particularly in, but not logically limited to, the study of harmful effects.

### Part B: The method of virtual controls

#### The better the counterfactual, the less need for randomization

On January 11, 1922, a 14-year-old named Leonard Thompson, who suffered with Type 1 Diabetes, was injected with an extract of insulin from animals’ pancreases [35]. On January 23, the patient began to receive a more purified extract. The patient’s blood sugar fell; he felt better; he survived. In February, six more patients had favorable responses to insulin. According to a fairly standard system of grading of research methodology, [36] this research would get a grade “C,” (where A is best) because the design is case series without randomization. Level 4 confidence would be attached to a recommendation that Type 1 diabetic patients be given insulin, where level 1 is represents most confidence.

Why was this research awarded the Nobel Prize rather than discounted as using “flawed methods?” Because the counterfactual, i.e. what would happen without this treatment was so readily predictable: approximately 100% chance of death. The better we can predict the counterfactual outcomes, the less we need randomized studies to see if treatment gives an incremental benefit. Glasziou and colleagues [37] compiled, in addition to insulin for diabetes, 18 other “examples of treatments whose effects had been widely accepted on the basis of evidence from case series or non-randomised cohorts”(p. 349), because the results of treatment were so good relative to past experience. The treatments include defibrillation for ventricular fibrillation, suturing of large wounds, ether for anesthesia, neostigmine for myasthenia gravis, and others.

The same point was made satirically in a tongue-in-cheek article [38] which reviewed randomized studies of “parachute use to prevent death and major trauma related to gravitational challenge.” Since no randomized trials were found, the authors suggested that the efficacy of parachutes is unproven, and that randomization-purists should volunteer for a randomized trial.

What if we can predict a counterfactual with nowhere close to 100% accuracy, but with “fairly good” accuracy? The less is our predictive accuracy, the more noise is in the system, and the greater the sample size we will need to estimate our treatment effect. But the basic reasoning -- predicting outcome from past experience and seeing how much the innovative treatment deviates from prediction -- is the logic behind virtual controls, just as in making conclusions about insulin and parachutes.

Much has been written about causal inference without randomization, including the use of case-control methods, covariate adjustment [39, 40] and propensity scores [41, 42]. These and other methods are outside the scope of the present discussion. Also outside the scope are “in silico” trials, predicting the effect of an intervention agent based on computer-intensive models of the mechanism of the intervention in question and the physiology of the recipient (see for example [43, 44]. The virtual controls method involves a much simpler technique of predicting outcomes without a certain intervention, using data from people who have not received the intervention. It is certainly not the only one of several methods of inference without randomization.

#### Counterfactual by virtual controls

The method of virtual controls uses a comparison of actual outcomes to counterfactuals, to predictions of what would have happened without the treatment. But the comparison outcomes are generated by statistical techniques, for example regression equations, derived from samples of untreated individuals, which incorporate important predictive variables. The detailed steps are as follows.

1. Cases are collected in which the outcome variable of interest is to be or has been measured. Knowledge about predictive variables is pooled, and those variables are measured to the extent that is possible or practical. Research participants do not need to consent to randomization -- only to the use of their data for science. These are cases where the intervention of interest has not been used -- perhaps because it has not been developed yet.
2. Statistical methods establish mathematical models predicting the outcome variable from the measurable predictors. In the simplest case, ordinary linear regression may be used for quantitative outcome variables and logistic regression for dichotomous outcome variables. The details of statistical alternatives are beyond the scope of this discussion. The precisions of the models are quantified, e.g. with the R-square statistic for regression.

To the extent that the ranges of any of the predictive variables are truncated, researchers are cautious about generalizing the equations to very different samples. But the data set can be pooled with other sets tapping into other parts of the variable ranges to permit more accurate prediction equations. For example, if initial “training samples” contain few with a smoking history, the sample can be purposely enlarged to include more smokers. The concerted and cooperative effort to progressively arrive at better prognostic models is one aspect that differentiates the method of virtual controls from simple covariate adjustment. A very important output of the research program is a gradually refined and revised set of prediction coefficients. As more data come in, the accuracy of prediction can be checked with independently collected data. If the model derived does not work well with a different data set, researchers search for the variables that account for the differences between the two samples, and add those variables to the predictive models; today’s confounder is tomorrow’s predictor. Data sets can be pooled and prediction models updated in an iterative process.

The process up to here is important in and of itself. It constitutes the quest for the most accurate possible prognosis. It aids understanding the reasons for favorable and unfavorable outcomes. It provides information on factors that may be subject to intervention. It enables clinicians to take the assembled coefficients, enter an individual patient’s predictive variables, and predict outcomes, together with an interval quantifying the confidence of the prediction. It enables scientists to be aware of how accurately predictable is the valued outcome in question.

3. When new treatment becomes available and appears promising, patients may be offered the treatment in a single arm trial, without randomization. The predictive variables are measured.
4. For each participant receiving the new treatment, the researchers plug into the modeling equation that participant’s values on the predictive variables. Thus the models produce either a quantitative outcome or the probability of an outcome (in the case of a dichotomous variable). The predicted outcomes are the virtual controls.
5. The participants receive the treatment that is being evaluated. Their actual outcomes are measured.
6. Effect sizes for the intervention, and confidence intervals for such, are generated by comparing the actual outcomes with those of the virtual controls. Confidence intervals may be put around mean outcome variables with and without treatment for quantitative variables, or around proportions of response with and without treatment for dichotomous variables.
7. As more data accumulate, prediction equations for outcomes both with and without the intervention are refined. The effect size estimates are upgraded.

With the output of such a research program, clinicians can gather information on predictive variables from individual patients, enter the values into the equations the research produces, and advise a patient as follows: “Given your data, the predicted outcome without this intervention is ____, and the predicted outcome with this intervention is _____.”

The outcome variables should include both favorable effects and adverse effects. The method can thus yield estimates of both risks and benefits with and without the intervention, so that both clinician and patient can be best equipped for decision making.

The “product” of this research method is not just a dichotomous yes or no decision on whether the intervention differs significantly from the comparison. It is not just an effect size estimate, for how much the intervention will add to the predicted outcome. It also includes a set of coefficients that enable prediction of outcome given the individual characteristics of the person, with and without treatment.

A similar strategy was proposed theoretically by Eichler et al. [45] in 2016; these authors called this strategy “threshold crossing.”

Let us examine the method of virtual controls with regard to the same five aspects with which we examined randomized designs.

#### Internal and external validity

Suppose, using the method of virtual controls, the intervention outcomes are significantly better than the outcomes predicted for the virtual controls. Can we be sure that the intervention caused those differences? No, because it is always possible that our intervention group differed from the group from which the prediction equations were derived (the training group), with regard to some key variable not measured, but nonetheless influential on outcomes. For example, we generate prediction equations for postintervention outcomes of depression versus happiness. We use preintervention mood scores, age, socioeconomic status, verbal ability, gender, support system, length of time depressed, history of trauma, family history, response to previous treatment, and so forth. But suppose we don’t measure motivation for improvement. And suppose it so happens that the group that signed up for the new intervention was exceptionally motivated to improve, and such motivation greatly influences the intervention’s usefulness. Selection, the classic threat to internal validity, could account for the differences. Even if we measure motivation, how do we know that some other unmeasured variable doesn’t account for the differences? It appears that the virtual control method has a fatal flaw.

But a similar fatal flaw applies if we imagine that instead that there is a randomized study. The group getting the intervention does significantly better than the control group. But now the relevant question becomes, “What will happen if the intervention is given to a different group of patients at my clinic?” Suppose the patients at my clinic happen to be much less motivated for improvement than the group in the randomized trial. Or suppose they differ on some other influential unmeasured variable. My inference from the randomized trial to predict outcomes with my clinic patients may be totally in error, because of the same “lurking variable” problem, despite randomization!

When the unmeasured variable is different across treated and untreated groups, we call the problem internal validity; when the variable is different across the subjects in the study and those to whom the study’s information is generalized, we call the problem external validity. But the basic problem wherein the effects of an unmeasured variable, or several of them, interfere with chain of inference, remains unsolved, and is not fully solvable by either alternative.

One advantage of the virtual controls program is that over time, investigators can listen to critics’ objections, develop measures for the lurking variables, and if they do prove predictive, include them in the revised set of equations. In the example above, investigators might try several different ways of measuring motivation for improvement, and one or more might be effective.

A second advantage of the virtual controls program is that even if the first group for which results are reported differs systematically from the training set, addition of data on more treated individuals would at least have a chance of correcting this. To continue our example, the first group of volunteers for the anti-depression method may have been unusually highly motivated. But as more and more people get treated, the average motivation of the total treated sample would probably tend to regress toward the mean for the population.

By contrast, if all we have to go on are randomized studies using people different from our clinic population, we might continue acting as if our treatment method were effective for our population, making the same error in perpetuity.

It is much easier to add more research subjects and revise conclusions with the virtual controls method, because the subjects are simply ordinary patients, getting the treatment they want, having variables carefully measured, and consenting for the data to be analyzed. Such people are omnipresent compared to the people who are willing to randomly be assigned to treatment versus no treatment.

#### Ethics

The virtual controls method eliminates the ethical issues that spring from making important treatment contingent on a random number generator. The research participants who do not receive an intervention in the virtual controls program have the intervention withheld because 1) the intervention has not been developed yet, 2) the participants choose not to get the intervention, or 3) the intervention is for some reason (including scarce resources) unavailable to those participants. The researchers do not need to purposely withhold a treatment they believe to be helpful, even life-saving, out of a need for rigorous research design.

#### Time and effort

The time and effort involved in obtaining research quality data should not be underestimated, no matter what the design. Non-blind ratings and measurements can be a decisive flaw for variables evaluated subjectively, such as ratings of depressed affect; it can be nonproblematic for variables such as death. Even keeping track of what intervention(s) were delivered, and for how long, can require careful effort sometimes lacking from ordinary medical or psychological or educational records.

Nonetheless, the method of virtual controls would be employed by interveners who ideally should keep careful measures with or without the research. The additional effort in making the measurements careful enough for research should enhance clinical practice. The additional effort should be much less expensive than conducting a randomized trial. In particular, the effort entailed in finding people willing to submit to randomization is eliminated.

With the coefficents the program derives and refines, a clinical group of any size, from a health care system to an individual practitioner, can measure the predictive variables for the patients under their care who receive some innovative method, or some quality-improvement method, and can test outcomes without needing to assemble a comparison group. For example, imagine that a mental health clinic has found that a set of coefficients predicting outcomes for child Oppositional Defiant Disorder treatment are reasonably accurate for their population. A psychoeducational parent training program is added to the standard intervention package; the new outcomes can be tested against the virtual controls generated by the coefficients. Intervention research can take place without asking patients to do anything other than get the best intervention and allow their data to be used. Thus the virtual controls method could result in a dramatic increase in meaningful intervention research that harnesses everyday clinical outcomes. This is one of the major benefits of using it more widely.

#### Time limits

As I previously noted, when people receive a certain treatment because they have contracted with the investigator to get whatever they are assigned, there are time limits on how long they should be expected to adhere to this assignment. With the method of virtual controls, there is not time pressure on the investigator to tell them, “Now you can receive whatever treatment you want” -- they are already receiving it. We must acknowledge, however, that without any influence of the investigators, and even with it, the combinations of treatments, and the lengths of time of adherence to various treatments that people will choose, will be quite heterogeneous. Some people will select themselves for longer intervention because of a positive response and wish for even more; others will select longer treatment because of a more chronic condition more refractory to brief treatment. The method of virtual controls will need to measure and take into account such differences. The study of long-term effects of long-term interventions is not easy. But it is at least possible, whereas maintaining randomly assigned conditions over years is often not possible.

#### The track record of the virtual controls method

The use of virtual controls in published research is in its infancy, but several studies have accumulated.

Jia et al. [46] studied the effectiveness of adjuvant therapy for prostate cancer. Statistical models were generated to predict time of progression free survival with non adjuvant-treated patients. The accuracy of prediction was checked with another independent sample, and found to be quite acceptable, despite cases for each sample having been drawn from the practices of different surgeons and several different years. Then the statistical models, together with the individual patient characteristics, were used to generate predictions for each patient receiving the experimental treatment in a single-arm trial. These predicted outcomes were the virtual controls. After the patients received the adjuvant treatment, their actual outcomes were compared with the predicted outcomes. The happy ending was that the adjuvant therapy appeared to work: “The observed PFS [progression free survival] significantly differed from the estimated PFS with chi-square= 19.3 and p value <0.0001.” (p. 6). The authors checked the results using historical controls, i.e. untreated patients selected so as to match the characteristics of the treated patients. The same conclusion was reached. The authors noted that the virtual control method has two advantages over historical controls: the virtual controls, generated by the treated patients’ actual numbers on prognostic variables, should resemble the patients more closely than historical controls for which the prognostic variables are only approximately the same. Second, there is more chance for bias to come in when researchers select matched subjects for historical controls, as contrasted to generating prediction equations from larger data sets.

Ketchum et al. [47] described another example of the use of virtual controls. The Seattle Heart Failure Model is a set of equations predictive of several important clinical outcomes in patients with heart failure, derived from some 20,000 patients. The model used at least 15 predictors: age, sex, ischemic etiology, heart failure class, ejection fraction, systolic blood pressure, and others. The researchers used the models derived with patients receiving medical therapy to predict one-year survival in 104 patients receiving medical therapy and a left ventricular assist device. The predicted 1 year survival rate in these very sick patients was 11%. With the assist device, however, the actual survival rate was 69%. This difference was highly statistically significant.

Switchenko et al. [48] checked the method of virtual controls using data from a trial of paclitaxel as adjuvant chemotherapy for breast cancer. A randomized trial [49] had tested paclitaxel plus the standard of care versus the standard of care alone. The authors made use of a preexisting statistical model for prediction of survival in breast cancer, given the standard of care. They refined that model with another training data set, and finally applied the model to data from the individuals in both arms of the previously completed randomized trial. The model successfully predicted the outcomes of the control arm. The difference between actual survivals of the experimental group and the virtual controls was similar to the effect found previously in the randomized study. The authors succinctly summarized the chief advantage of virtual controls: “We were able to reach a conclusion similar to that reached in the actual study about the benefits of paclitaxel without the need to enroll thousands of control patients to receive standard of care ... alone.”(page 9).

Neal et al. [50] described a method of generating “untreated virtual controls” for patients with glioblastoma multiforme, a highly invasive brain tumor. In this case, rather than using untreated individuals to generate the prediction of untreated course, the investigators used the rate of progression of tumor during the interval between two MRI’s taken before treatment began. The mathematical models were complex, taking into account the three-dimensional characteristics of the tumors. Simulation of tumor progression projected progression-free-survival and overall survival without treatment. Comparing that prediction to the actual numbers after the first radiation treatment allowed a “days gained” measure, the difference between actual outcome and virtual control outcome for each patient. These authors found that the days gained predicted future survival.

Carrigan and colleagues [51] remarked upon the increasing reliance on single arm trials for oncology drug development. They observed that “Curated electronic health record (EHR) datasets are now large enough, with sufficient clinical detail, to create contemporaneous external control (EC) groups.” (p. 370) These investigators examined 8 randomized studies of lung cancer treatment, and compared effect sizes from RCTs with those computed using the “external controls.” They found that the “EC-derived hazard ratio estimates aligned closely with those from the corresponding RCT with one exception. Comparing log HRs among all RCT and EC results gave a Pearson correlation coefficient of 0.86.”(p. 369).

An exploration of virtual controls in the mental health arena was with the Strengths and Difficulties Questionnaire, a widely used broad spectrum short measure of psychological symptoms and functioning in children and adolescents. Ford et al. [52] used linear regression to derive a prediction of the “total difficulties” subscale 4 to 8 month postscores, from three subscales of the prescores on the same measure, using a sample of 609 children. With three regression coefficients and a constant term, the postscores could then be predicted for other samples. The actual postscores minus the predicted ones were called the “added value” scores by this group. The researchers then used this approach with the treated children from a randomized trial of a parenting program; the effect size for treatment when compared to the computationally derived controls was 0.36. In the actual randomized trial, the effect size for the difference using randomly assigned real human untreated controls had been 0.37. Although the confidence interval for the effect sizes suggests that it was partly coincidental that they landed so very close together, the experience strengthened confidence that the added value scores derived from the regression equations could enable single arm research without randomly assigned no-treatment controls.

Interestingly, this study reported that the added value scores did not share a significant amount of variance with a number of other predictors, including: “type and severity of diagnosis, age, gender, intelligence, physical health, maternal educational level, maternal anxiety or depression, family type, family function, family size, income, housing tenure and neighbourhood characteristics,” whereas these variables explained about 36 and 24%, respectively, of the variance in the prescores and postscores. Thus while this set of variables influenced the *levels* of the child’s functioning, they were not needed for a prediction of the *change* in the child’s functioning. The influence of these variables was apparently factored into the prescores sufficiently that the prescores could predict well enough without them.

An exception to the enthusiastic endorsement of virtual controls came in a study by Hansen et al., [53] who explored the feasibility of virtual controls for studies on the prevention of use of alcohol, marijuana, and cigarettes in adolescents. These authors created an algorithm for prediction of use of these specific substances by integrating the results from eight longitudinal studies from 1980 to 2010. The algorithm was then applied to the data from two randomized controlled trials, to see how well the model predicted the preintervention prevalence of the use of the substances, and the progression of substance use in the control group. The prediction of preintervention substance use was very close; the prediction of progression over time for the control groups of the randomized studies was good for alcohol; for marijuana and cigarettes the results were mixed -- not precise enough to justify single-arm studies subsequently. It is plausible that the prediction of a specific behavior, such as use of a certain substance, which is susceptible to fairly rapid cultural shifts in peer influence, is more difficult to predict than such variables as tumor progression or overall psychological functioning.

The use of data generated from computer-intensive modeling [54], and the use of data from actual clinical practice rather than interventions delivered only because of a study (called “Real-World Evidence”), have each been the subject of recent guidelines from the U.S. Food and Drug Administration. I include as the final example of something that sounds close to the use of virtual controls, the following from the FDA Guidance on Real World Evidence [55].

“A manufacturer approached FDA during the development of a next generation medical device that had substantial technological changes from previous iterations of that specific device and other similar devices from other manufacturers. FDA determined that clinical evidence was needed to support an approval decision for this device modification. A registry exists that captures RWD [real world data] on all uses of medical devices with a similar intended use. The manufacturer designed a clinical study that compared the use of the new device to a non-randomized concurrent control group derived from the registry. The existing registry was evaluated by FDA and the manufacturer according to the factors cited in this guidance and was found to provide sufficiently relevant and reliable RWD on the control population, such that the manufacturer did not have to collect additional data from these patients or influence the course of their clinical care in any way.” (p. 19).

In all these reports, including the FDA guidance, the authors seem obviously aware that they are treading new ground in intervention testing methodology by deviating from gold standard randomized trials.

## Conclusions

Let us conclude by enumerating aspects of research questions and situations that might dispose one toward randomized designs, and toward virtual control group designs.

We would tend to favor randomized designs the more the following conditions hold:

1. It appears likely that differences between groups in the trial itself would be more important a source of bias than differences between the research sample and the population to which the results are generalized. In other words, internal validity appears more important to protect than external validity. As an example, there would be big differences between those who self-select for treatment and those who don’t, but not big differences between the research participants and the patients for which inference is desired.
2. The therapeutic agent has its effects quickly enough that the group not receiving it can benefit from it if it proves efficacious. For example, a test for an anti-migraine drug lasts a matter of weeks, and the placebo subjects can benefit from it long after the trial is over.
3. The effects of withholding treatment are not permanent. For example, the therapeutic agent is not life-saving or permanently life-altering.
4. The research community has not accumulated data permitting prediction of outcomes without the intervention, and obtaining that data will take much longer than assembling a group for a randomized trial.
5. The intervention is an expensive one; there are funds available only for a limited number of interventions; the fairest way to select individuals for intervention is a random lottery.
6. Blind conditions are feasible enough that resentful or sorrowful demoralization about group assignment does not confound the differences between groups.
7. There is a widespread belief that the intervention is useful, but the investigators are skeptical, and are at “equipoise” regarding the intervention, suspecting that the null hypothesis is true.
8. The conditions to which participants are randomized are a new method and an active, efficacious method; the investigators suspect equal efficacy for each. The new method is less expensive or easier to deliver or has some other advantage that does not impinge upon the participants’ welfare. Thus the study is a non-inferiority trial.
9. The research participants fully understand randomization, will be satisfied with participation regardless of group assignment, and have not signed consent forms that are too legalistic for them to read carefully and understand.

We would tend to favor the method of virtual control the more the following conditions hold:

1. There is reason to believe that the effect size of the intervention is positive, large and important (e.g. preservation of life). This condition simultaneously makes it less ethical to withhold treatment, because of responsibility to placebo subjects, and less necessary to withhold treatment, because the signal is more likely strong enough to be seen through the noise, even if the treated group has some differences from the training group for the virtual controls.
2. The search for accurate predictors of outcome is deemed valuable in itself.
3. There is a wish to predict treatment response with a more heterogeneous group of patients than a randomized trial could enroll; the training sample for the virtual controls can be more diverse, because the people in the sample are not limited to those volunteering for randomization.
4. There are good data on prediction of outcome derived from training samples studied before the development of the intervention.
5. Time is of the essence is testing the intervention, and results can be achieved more quickly by having a single arm rather than a double arm trial. (For example, because many more people are willing to volunteer to get the experimental treatment than to participate in a randomized trial of it.)
6. The intervention is a long term one, for example lasting several years, and the virtual controls design eliminates the ethical and some of the practical problems of maintaining treatment conditions.
7. It is not possible for research participants to be blind, as for example in most psychotherapeutic or psychoeducational interventions, and participants are likely to prefer one condition to another. Thus the virtual controls method obviates the resentful or sad demoralization from randomization to non-preferred treatment.
8. Investigators and/or participants, for any other reason, value participants’ ability to choose their own treatment.
9. Which treatment the participants elect to receive is not highly correlated with unmeasured or unmeasurable variables affecting outcome.
10. There is a wish to take advantage of “real-world data” and not to waste this source of information.
11. There is a wish to individualize clinical decision-making by predicting outcomes with and without treatment for particular patients, using the statistical models generated.

Future research on this topic should include more studies in which the results of randomized trials are compared with those obtainable through virtual controls, as in some of the examples I presented. Systematic compiling of the extent of accuracy in prediction of important outcome variables would be of major benefit to the use of this method, as would systematic compilation of the equations derivable from current data that allow time 2 outcomes to be predicted from time 1 data and intervening events. Simulation studies may be quite informative in explicating the conditions under which the virtual controls method actually gives a more accurate decision than randomization, and under which the opposite is true. One possible question for simulation might be: how accurate does prediction have to be, before the virtual controls method yields correct decisions? Further exploration of the statistical techniques most effective in carrying out the method of virtual controls would also be enlightening (with both efficiency and simplicity in analysis being afforded weight).

I write this in the era of the COVID-19 pandemic. It is tempting to say that intervention research design has taken on new importance of late. But the ways in which we determine what actions help and hurt people, and how much good or harm these actions do, have always been important, and always will be. Perhaps employment of the virtual controls program will turn out to provide some incremental improvement in our ability to decide how best to help one another.

## Data Availability

All information reviewed is available in the referenced articles and books.

## Abbreviations

CAMS: Child and Adolescent Anxiety Multimodal Study
EBM: Evidence Based Medicine
EC: External Controls
EHR: Electronic Health Record
FDA: Food and Drug Administration
HR: Hazard Ratio
MRI: Magnetic Resonance Imaging
NHS: Nurses’ Health Study
OCD: Obsessive-Compulsive Disorder
PFS: Progression Free Survival
RCT: Randomized Controlled Trial
RWD: Real World Data
TADS: Treatment for Adolescents with Depression Study
POTS: Pediatric OCD Treatment Study
WHI: Women’s Health Initiative
